# Adherence to self-managed exercises for patients with persistent subacromial pain: the Ad-Shoulder feasibility study

**DOI:** 10.1186/s40814-021-00767-6

**Published:** 2021-01-25

**Authors:** Daniel H. Major, Margreth Grotle, Chris Littlewood, Jens Ivar Brox, Dagfinn Matre, Heidi V. Gallet, Yngve Røe

**Affiliations:** 1 Faculty of Health Science, Oslo Metropolitan University, Oslo, Norway; 2 Research and Communication Unit for Musculoskeletal Health, Oslo University Hospital, Oslo, Norway; 3 Department of Health Professions, Manchester Metropolitan University, Manchester, UK; 4 Department of Physical Medicine and Rehabilitation, Oslo University Hospital HF, Oslo, Norway; 5 Department of Work Psychology and Physiology, National Institute of Occupational Health, Oslo, Norway; 6 Diakonhjemmet Hospital, Oslo, Norway

## Abstract

**Background:**

Exercise is recommended for patients with subacromial pain. It has been suggested that good exercise adherence improves clinical outcomes. Despite this, little attention has been paid to the need for behavioural frameworks to enhance adherence to home exercise programmes for patients with subacromial pain.

**Methods:**

A feasibility study with pre-post design was used. Participants aged > 18 years, with subacromial pain, who had received conservative treatment during the past 6 months, were recruited. The Ad-Shoulder intervention consisted of 1–5 individual sessions provided over 3 months and was based on 5 self-management skills, which aimed to enhance the patients’ self-efficacy and adherence to self-managed exercises. The primary objectives were assessed according to predefined progression criteria: (1) the recruitment rate (10 patients enrolled within 12 weeks), (2) follow-up rate (≥ 80% on all self-reported measures), (3) objective physical activity measures (≥ 80% of participants would contribute valid data at each time point), (4) adherence with the self-managed exercises (≥ 80% of the participants would adhere to ≥ 80% of the assigned home exercise programme), (5) fidelity of the delivery of the intervention (the therapists delivered the intervention according to the protocol) and (6) adverse events (< 30% would report adverse events (including mild)). The results were reported using descriptive statistics.

**Results:**

Eleven patients were recruited during 16 weeks. Ten patients completed the self-reported measures at baseline and week 12. Objective physical activity measures were successfully obtained for 100% (11/11) at baseline, 64% (7/11) at week six and 82% at week 12. Fifty-five percent (6/11) of the participants satisfactorily completed at least 80% of their home exercise programme. All sessions were delivered according to the protocol. None of the patients reported any adverse events.

**Conclusions:**

Objective physical activity data measures at baseline and week 12, follow-up, the physiotherapists’ fidelity to the intervention and adverse events met our pre-specified progression criteria. Recruitment and adherence to the self-managed exercise programme were both below the anticipated level. Further intervention development is necessary to understand whether adherence to the self-managed exercises could be enhanced and additional methods of recruitment would need to be considered, including additional recruitment sites, in any planning for a future main trial.

**Trial registration:**

ClinicalTrials.gov, NCT04190836, Registered December 9, 2019—retrospectively registered

**Supplementary Information:**

The online version contains supplementary material available at 10.1186/s40814-021-00767-6.

## Key messages regarding feasibility

1. What uncertainties existed regarding the feasibility?
  We wanted to investigate the recruitment rate, follow-up rate, objective physical activity assessment, adherence to the self-managed exercises, fidelity and adverse events of the Ad-Shoulder intervention.
2. What are the key findings?
  Objective physical activity measures, follow-up, the physiotherapists’ fidelity to the intervention and patients reporting adverse events (including mild) were judged to be feasible.
  The feasibility of recruitment and adherence to the self-managed exercise programme were below the expected level.
3. What are the implications of the findings for the design of the main study?
  Further intervention development is necessary to understand whether adherence to the self-managed exercise could be enhanced.
  Include additional recruitment sites to optimise recruitment.

## Background

Shoulder pain is a prevalent and often long-lasting musculoskeletal disorder [1, 2]. Shoulder pain disorders are common in clinical practice, with an incidence rate of about 10 per 1000 in primary care [3, 4]. The impact on people with shoulder pain disorders is multi-dimensional including pain, activity limitations, social restrictions, sleep disruption, cognitive dysfunction, emotional distress and other pathophysiological manifestations [5]. Subacromial pain is the most common shoulder diagnosis, accounting for up to 70% of the cases [3, 6, 7].

Exercise is recommended as one treatment for patients with subacromial pain [8–11]. Although research suggests that exercise including some level of resistance and maintained for at least 12 weeks might be important prescription parameters, there remains a lack of knowledge about the optimal type- and dosage of exercises [12]. There is evidence to suggest that self-managed home exercises confer similar outcomes to supervised exercise programmes [12, 13]. In patients with chronic musculoskeletal pain, it has been suggested that there is an association between exercise adherence and improvements in clinical outcomes [14–16]. A Cochrane review on interventions to improve adherence to exercise in patients with chronic musculoskeletal pain concluded that individualised exercise therapy and self-management techniques may enhance exercise adherence [17]. There is also moderate quality evidence that behaviour change techniques, such as social support, goal setting, demonstration of behaviour, graded tasks and self-monitoring of behaviour, may improve exercise adherence among patients with persistent musculoskeletal pain [18, 19]. Despite this, behavioural frameworks to enhance adherence to home exercise programmes have been little implemented in current trials on shoulder pain [20].

In order to improve adherence to exercises in patients with subacromial pain, we developed a personalised supported self-management intervention (the Ad-Shoulder intervention). In order to assess the feasibility and acceptability of an intervention, it has been recommended to run a feasibility study, before commencing a main randomised controlled trial (RCT) [17–19]. Feasibility studies are designed to answer whether the study protocol can work and allows for modification of the protocol before commencement of the main trial [19, 21–23]. Hence, the aims of this study were to assess the feasibility of the data collection procedures and the acceptability of the Ad-Shoulder intervention.

## Methods

### Trial design

The feasibility study had a single-group pre-post intervention design. The trial is reported according to the Consolidated Standards of Reporting Trials (CONSORT) 2010 statement: extension for pilot/feasibility studies (Additional file 1) [24]. The trial protocol was registered with the ClinicalTrials.gov registry (NCT04190836, December 9, 2019) and approved by the Norwegian Regional Ethical Review Board (ref. no. 2017/355, April 21, 2017).

### Participants

The inclusion criteria were as follows:

- Adults (aged > 18 years) with shoulder pain located in the upper arm
- Previously received conservative care due to subacromial pain but still seeking primary or secondary care during the past 6 months

The exclusion criteria were as follows:

- Bilateral shoulder pain
- Clinical presentation consistent with a frozen shoulder diagnosis (< 50% external rotation compared to contralateral side) [8].
- Patients who have received surgical treatment due to shoulder problems
- Pregnancy
- Patients with insufficient Norwegian language skills
- Serious psychiatric disorder

### Recruitment and enrolment

Recruitment for this feasibility study was conducted by two practitioners (1 general practitioner and one physiotherapist) across two sites from November 2017 to January 2018 and May 2018. The two outpatient clinics were located in Oslo, Norway, of which one in primary care (Oslo Metropolitan University) and the other in secondary care (Diakonhjemmet Hospital). Potential participants were identified when seeking care for shoulder pain. The potential participants were pre-screened by a physiotherapist or a medical doctor at one of the recruitment sites. The final enrolment was conducted by two researchers (DHM and HVG) that would be providing the intervention, and eligibility for the study was re-checked. Informed consent was provided by all participants at inclusion after being provided oral and written information. During the recruitment process, it was deemed necessary to include additional eligibility criteria because the physiotherapists conducting the assessment at the second screening diagnosed patients with other diagnosis than subacromial pain. Therefore, clinical signs of a total rotator cuff tear, clinical signs of instability, clinical signs of a cervical syndrome, clinical signs of AC joint arthritis and reasons to suspect systemic pathology including inflammatory disorders were added to the exclusion criteria [8]. Clinical signs of subacromial pain was added to the inclusion criteria to confirm the diagnosis [8].These criteria are consistent with the British Elbow and Shoulder Society (BESS) guidelines [8].

### The Ad-Shoulder intervention

The Ad-Shoulder intervention was developed by DHM and YR on the basis of the self-managed single exercise programme by Littlewood et al. [25–29] and informed by recent research on subacromial shoulder pain [10, 12] and adherence to exercises for patients with persistent musculoskeletal pain [30]. The behavioural component of the Ad-Shoulder intervention was based on the self-management framework, provided by Lorig and Holman [31], A key component in this framework is to target patients’ self-efficacy, defined as the confidence to perform a specific task or behaviour [32]. In Bandura’s Social Cognitive Theory, the person’s perceived self-efficacy is thought to mediate behaviour change [32], which in this self-management intervention is closely linked to adherence to home exercises and physical activity. In the self-management framework suggested by Lorig and Holman [31], the ability to self-manage is achieved based on learning five core self-management skills. These are problem-solving, decision-making, resource utilisation, the forming of a patient/health care provider partnership and taking action. These core self-management skills are elaborated according to the intervention in Appendix 1. The intervention consisted of 1–5 individual sessions over 3 months, where the first session had a duration of 1 h and the following sessions about 45 min. The self-management strategy emphasises dynamic, progressively loaded exercises for the shoulder (Table 4). To enhance exercise adherence, we used behaviour change techniques such as social support, goal setting, demonstration of behaviour, graded tasks and self-monitoring of behaviour (Appendix 1). For specific content reporting of the home based exercises, we have followed the Certificate on Exercise Reporting Template (Additional file 2) [33, 34]. The participants had the option to contact the physiotherapist by phone, text message or e-mail for advice for up to 12 weeks, the duration of the intervention. Patients were allowed to continue with their usual medication, but were asked not to receive other treatment. The intervention was delivered by a PhD student (DHM) and a master student (HVG), both qualified physiotherapists’, with a special interest in shoulder pain and at least 4 years’ experience with assessment and management of musculoskeletal pain conditions.

### Measurements

The participants filled in a self-reported questionnaire at baseline (right before the first consultation) and at week 12. In addition, three self-reported measures (Pain Self-Efficacy Questionnaire 2-item, Numeric Pain Rating Scale and Self-Efficacy) were collected repeatedly, and data on objective physical activity was collected with accelerometers one week before baseline, at week 6 and at week 12.

The questionnaire package consisted of sociodemographic variables (age, sex, duration of shoulder pain, education level, work status, relationship status, smoking status, height and weight) and patient-reported outcome measures. An overview of the self-reported measures and time points is provided in Table 1 and a description of these are provided in Appendix 3.

**Table 1:** Overview of self-reported measures and the time points for the measurements

	Baseline	Week 6	Week 12
Shoulder Pain and Disability Index [35]	X		X
Patient Specific Function Scale [36, 37]	X		X
Numeric Pain Rating Scale [38]*	X	X	X
Self-efficacy [39]*	X	X	X
Pain Self-Efficacy Questionnaire 2-item [40]*	X	X	X
Work ability index [41]	X		X
EQ-5D-5L [42]	X		X
Bergen Insomnia Scale [43]	X		X
Kinesiophobia [44]	X		X
Patient-reported physical activity [45]	X		X
Hopkins Symptom Checklist 25 [46]	X		
Expectations of recovery [47]	X		
Global Perceived Effect Scale [48]			X
Adverse events			X

*Also measured with repeated measurements (total of 30 measures)

The participants also responded to SMS text messages containing three measurements (Numeric Pain Rating Scale, Self-efficacy and Pain Self-Efficacy Questionnaire 2-item) before, during and after the intervention period to explore the process of change on an individual level. During the first week pre-treatment phase (A), 3 text messages including the three measurements were collected. During the 12-week treatment phase (B) the text messages were collected twice every week (total of 24), and during the one week post-treatment phase (A) the text messages were collected 3 times.

Physical activity was objectively measured, using four accelerometers (AX3, 3-axis Logging Accelerometer, Axivity, UK) attached to the chest, upper arm, hip and wrist for 3 consecutive days 1 week before baseline, at week 6 and at week 12. This accelerometer provides information about movement and enabled us to objectively measure the amount of general physical activity (minutes of ≥ moderate activity, defined as metabolic equivalent (MET) value ≥ 3).

### Feasibility outcomes and progression criteria

1. The recruitment rate—measured by how many people that were eligible and how many people that were recruited per week

Progression criteria: Ten participants would be enrolled in the study within a 12 week period.

2. Follow-up rate—measured by the percentage of participants who were followed up successfully until the three months follow-up

Progression criteria: Follow-up rates ≥ 80% for the questionnaire at 12 weeks and the repeated measures.

3. Feasibility of actigraph assessment of physical activity—measured by percentage of participants with valid data at each time point (baseline, 6 weeks and 12 months). Validity of data was defined as successful measurement of a participant’s physical activity for at least 20 h day for three consecutive days.

Progression criteria: At least 80% of participants would contribute valid physical activity (actigraph) data at each time point.

4. Adherence with the self-managed exercises—measured by percentage of patients maintaining at least 80% adherence to the self-managed exercises measured by self-reported exercise logbook.

Progression criteria: At least 80% of the participants would adhere to at least 80% of the assigned home exercise programme (self-reported).

5. Fidelity of the delivery of the intervention—measured by whether the physiotherapist delivered the components of the intervention or added other components to the intervention using a physiotherapist completed logbook at 3 months.

Progression criteria: The therapists would deliver the intervention according to the protocol (100%).

6. Number and nature of adverse events—measured by self-report questionnaire.

Progression criteria: No more than 30% would report adverse events (including mild), such as increased short term pain with home exercises.

7. Data collection procedure—assessed by exploring data from the self-report outcomes with respect to missing data and scoring pattern at baseline (floor/ceiling effect, median, variation).

In terms of decision-making against the progression criteria, should we have fallen below/above any of these rates in our feasibility study we would consider whether protocol modification or close monitoring during a main RCT would address any failure to meet these criteria, or decide that the main RCT would not be feasible [19].

#### Sample size

As this is a feasibility study and inferential statistics were not calculated there was no need to achieve a desired power to detect an effect. The research team decided that 10 participants would be sufficient to give a preliminary understanding of the feasibility of data collection procedures and acceptability of the Ad-Shoulder intervention.

#### Blinding

Due to practical reasons, the patients and clinicians were not blinded to the treatment and the researcher conducting the analyses (DHM) was one of the therapists in the intervention group.

#### Statistical methods

Descriptive statistics were used to assess the feasibility objectives and the patient-reported outcomes using SPSS (version 25, IBM, Armonk, NY, USA) and Microsoft Excel (2016). Due to the low number of participants, the continuous variables were presented as median and interquartile range or min–max values.

## Results

### Sample characteristics

The included participants had a median age of 48 years (IQR 15), seven of the 11 participants were female, and the median duration of shoulder pain was 18 (IQR 15) months. The baseline characteristics of participants are provided in Table 2.

**Table 2:** Patient demographics and patient characteristics (*n* = 11)

Variables	*n*	Median (IQR)
Gender, woman	7	
Age		47 (14)
Body Mass Index		22.8 (2)
Smoking status, no	11	
Duration of symptoms (months)		18 (15)
Hopkins Symptom Checklist 25		1.2 (0.6)
Working status		
Working	9	
Sick leave	2	
Educational level		
Primary school	0	
High school	0	
Higher education ≤ 4 years	4	
Higher education > 4 years	7	
Relationship status		
Married/in a relationship	7	
Divorced	2	
Widow/widower	0	
Single	2	
Expectations of recovery		
Complete recovery	4	
Much improved	6	
Slightly improved	1	
No change or worse	0	

### Recruitment capability

**Fig. 1:**
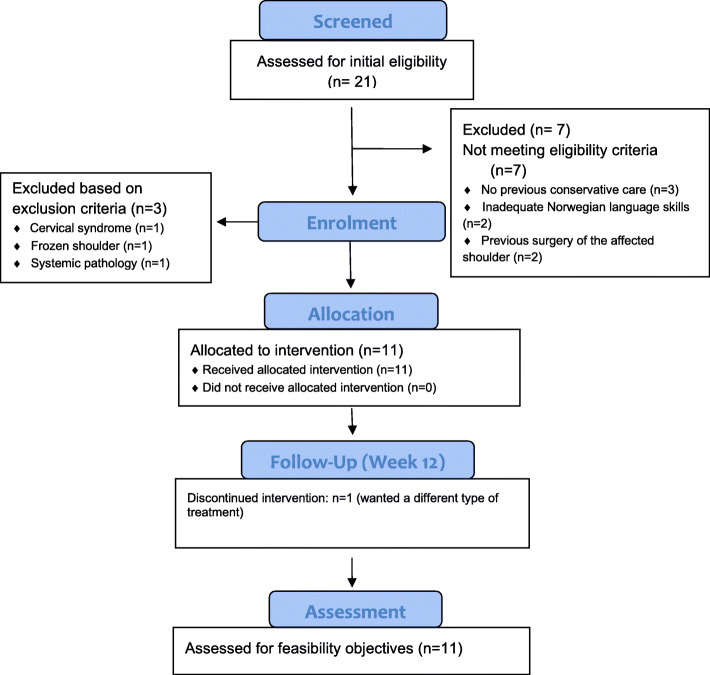
CONSORT 2010 flow diagram. Design and flow of participants through study

Flow of participants through the study and reason for exclusion is presented in Fig. 1. The 11 patients were included during a time-period of 16 weeks (November 2017–January 2018 and May 2018). We had to use 4 more weeks than what was anticipated before commencement of the study.

### Follow-up

Ten of the eleven participants completed the questionnaire package at baseline and week 12. For the repeated measures, 313 of 330 (95%) were collected. There were 17 occasions of a reminder being sent; of these, all except two were successful. Ten of the eleven included patients delivered complete exercise logbooks at week 6 and week 12.

### Objective assessment of physical activity

Concerning the actigraph measurements, objective physical activity was successfully obtained for 100% (11/11) of the participants at baseline, 64% (7/11) participants at week 6 and 82% (9/11) participants at week 12. The reasons for the missing data were time constraints (one participants at week 6), being abroad on holiday (one participant at week 6), skin eczema (one participant at week 6 and 12), skin irritation (one participant at week 6) and one patient dropped out of the study (one participant at week 12). The calculated time-consumption for administration of the four actigraph sensors was a total of 1 h per patient at each time point, excluding data analysis. In the analysis of the data, we were able to obtain a good impression of the general physical activity level, using only one accelerometer (wristband).

### Adherence

The included patients reported a median of 3 sessions (range 1–5) during the 12-week intervention period. With respect to treatment adherence, all patients met to the scheduled appointments.

Based on information obtained from the exercise log, median adherence was 86.5% (range 47–97). Fifty-five percent (6/11) of the participants satisfactorily completed at least 80% of their home exercise programme. The four patients that had self-reported adherence to exercises < 80% reported the main barriers to adherence to be time constrains (*n* = 2), forgetfulness (*n* = 1) and one patient was not able to do the exercises due to periods of sickness (*n* = 1).

### Fidelity

According to the logbook completed by the physiotherapists that delivered the Ad-Shoulder intervention, all sessions were delivered as planned. One of the therapists (HVG) contacted one of the developers of the intervention (DHM) twice via telephone to get advice and to make sure she adhered to the treatment protocol.

### Adverse events and other treatment

**Fig. 2:**
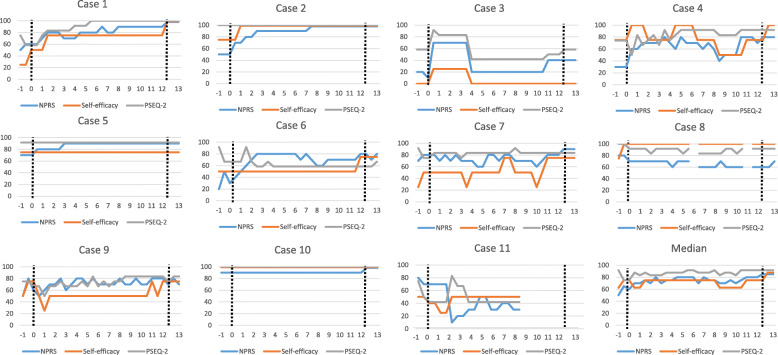
Repeated measures at 30 occasions for the 11 patients converted to a value of 0–100, where 100 indicates a better score. Measurement number 1–3 were 1 week before the intervention started, 4–27 were done twice weekly during the intervention period (12 weeks), and 28–30 were 1 week after the intervention. NPRS, Numeric pain rating scale; PSEQ2, Pain Self-Efficacy Questionnaire 2 item; Self-efficacy, “How confident have you felt about managing your shoulder pain by yourself?”

None of the patients reported any adverse events at 12 weeks follow-up. One patient (case 4 see Fig. 2) reported that she had received other treatments during the intervention period (manual therapy and one cortisone injection).

### Patient-reported measurements

The results of the patient-reported measures are displayed in Table 3. The median change from baseline to week 12 in the SPADI was 18 (range − 0.8 to 53). The data from the repeated measures (NPRS, PSEQ-2 and general self-efficacy) are presented for every individual and the group in Fig. 2. At baseline the median PSEQ-2 score was 9 (range 7–12), where three of the 11 patients scored the maximum score. Five of the included patients felt very confident in being able to manage their shoulder pain by themselves at baseline (general self-efficacy). The median change from baseline to week 12 in PSEQ-2 and general self-efficacy was 0 (range − 1 to 3) and 1 (range 0–3), respectively.

**Table 3:** Measurements at baseline and 12 weeks follow-up (*n* = 11)

	SPADI week 0/12	PSFS week 0/12	NPRS week 0/12	Kinesiophobia week 0/12	WAI week 0/12	Self-Efficacy week 0/12	PSEQ-2 week 0/12	BIS week 0/12	EQ5D-5L week 0/12	Physical activity week 0/6/12	GPE week 12
Case 1	56/3	5/9.7	5/0	1/1	5/9	4/1	9/12	22/13	0.67/1	37/48/23	1
Case 2	19/0	2.7/10	5/0	6/0	10/10	2/1	12/12	6/0	0.82/1	31/-/52	1
Case 3	67/46	0.3/2.3	9/6	2/1	4/7	5/5	7/7	35/32	0.73/0.52	58/-/-	4
Case 4	43/32	3/5.3	7/3	7/4	9/10	2/2	9/11	9/11	0.73/0.75	293/358/215	3
Case 5	20/5	6.3/6.3	3/1	5/1	10/10	2/2	11/11	10/4	0.86/0.86	120/-/217	2
Case 6	57/21	8.7/8.3	8/2	8/7	7/8	3/2	9/8	25/16	0.59/0.73	217/323/323	2
Case 7	19/5	6.3/7.7	3/1	7/1	8/9	4/2	11/10	10/4	0.79/0.86	216/204/158	3
Case 8	22/23	2.7/3.3	2/3	0/0	5/6	2/1	12/11	7/2	0.64/0.82	343/617/348	3
Case 9	38/22	3.3/7.3	5/3	2/2	6/7	3/2	9/9	12/6	0.67/0.79	120/137/202	2
Case 10	22/3	5/10	2/0	0/0	10/10	2/1	12/12	10/9	0.86/1	132/84/41	1
Case 11	65/-	7/-	6/-	7/-	7/-	4/-	8/-	14/-	0.73/-	110/-/-	-
Median (range)	38 (19–67)/21 (0–46)	3.3 (0.3–8.7)/8 (2.3–10)	5 (2–9)/2 (0–6)	3.5 (0–8)/1 (0–7)	7 (0–10)/9 (6–10)	2.5 (2–5)/2 (1–5)	9 (7–12)/11 (7–12)	10 (6–35)/6 (2–32)	0.73 (0.59–0.86)/0.84 (0.52–1)	132 (31–343)/170 (48–617)/158 (23–323)	2 (1–4)
Median change week 0/12 (*n* = 10)	18 (− 1 to 53)	2.2 (− 0.4 to 7.33)	2.5 (− 1 to 6)	1 (0–6)	1 (0–4)	1 (0–3)	0 (− 1 to 3)	6(− 2 to 9)	0.13 (− 0.21 to 0.33)	14 (− 91 to 107)	-
Missing data	-/1	-/1	-/1	-/1	-/1	-/1	-/1	-/1	-/1	-/4/2	1
Floor effect*	-	-	-	-	-	-	-	-	-	-	-
Ceiling effect**	-	-	-	2	3	-	3	-	-	-	-

SPADI, Shoulder Pain and Disability Index (0–100, 100 worst score); PSFS, Patient Specific Function Scale (0–10, 0 = unable to perform activity); NPRS, Numeric pain rating scale (0–10, 10 = most intense pain imaginable); Kinesiophobia (0–10, 10 = very much fear); PSEQ2, Pain Self-Efficacy Questionnaire 2 item (0–12, 0 = not at all confident); Self-efficacy, “How confident have you felt about managing your shoulder pain by yourself” (1–5, 5 = not at all confident); BIS, Bergen Insomnia Scale, 0–42 (42 = worst score); WAI, Working Ability Index, 0–10 (0 = cannot work at all); EQ5D-5L, 1 = perfect health; physical activity, minutes of moderate physical activity (MET ≥ 3) during three consecutive days measured using an accelerometer; GPE, global perceived effect scale, 1–6, 1 = very much better, 6 = very much worse

*Number of patients with the worst score at baseline

**Number of patients with the best score at baseline

## Discussion

The present feasibility study demonstrated that the Ad-Shoulder protocol was feasible with respect to follow-up rate and objective physical activity measurements at baseline and week 12. Furthermore, the Ad-Shoulder intervention was acceptable in terms of intervention fidelity and no adverse events were reported. In contrast, the recruitment rate and the patients’ adherence to the self-managed exercise programme were below our predefined progression criteria.

The Ad-Shoulder intervention was designed to enhance adherence to the self-managed exercises through a behavioural framework. One of the main challenges in light of progressing to a main RCT was that only six of 11 participants had an acceptable adherence rate to self-managed home exercises, despite that they had excellent attendance to physiotherapy sessions and that the physiotherapists had good fidelity to the Ad-Shoulder protocol. Our finding suggests that additional initiatives need to be taken in order to enhance adherence to the self-managed exercise programme between the consultations with the physiotherapist(s). Qualitative research to explore barriers and facilitators to adherence to the self-managed exercises might help the research team to develop strategies to enhance the adherence rate before commencing on future clinical trials [49, 50].

The lack of recruitment capability in the present study provides another challenge in the perspective of conducting the main RCT. The reason for the low recruitment rate was due to a low number of eligible participants. However, all eligible participants consented to participate in our study. This is similar to a Norwegian RCT among patients with subacromial pain, where only two out of 141 (1.5%) declined to participate [51]. In a RCT with a calculated sample of approximately 150 patients, the present results indicate a recruitment period of 218 weeks (over 4 years) and screening of approximately 300 potentially eligible patients. To be able to conduct a large multicentre trial with several involved people, we will require additional funding for necessary staff involved in the different phases of the trial (recruitment, randomisation, delivery of the treatment, follow-up procedures and analyses).

The change in the clinical outcomes during the 12 weeks of follow-up demonstrated that most participants improved. The median change on the SPADI of 17 points is slightly above the estimate for a minimal important change, which has been reported to be 8–13.2 [52]. However, it is important to interpret these findings carefully, as we cannot distinguish between effects that might have occurred due to the natural course of the condition, regression to the mean, placebo or the Ad-Shoulder intervention. Similar improvements were seen in pain intensity during the 12 weeks of follow-up, whereas in the two potential mediator measurements (self-efficacy and PSEQ-2) for the future RCT, little change occurred. The lack of change in pain self-efficacy and general self-efficacy might be due to the particular measurements we used, due to the high scores among many of the included patients at baseline or that the intervention is not performing as we assumed that it would. This finding warrants further exploration of whether pain self-efficacy and general self-efficacy are the most relevant mediators to explore in a future main RCT.

### Strengths and limitations

A strength of this study is that we used important benchmarks that have previously been recommended to assess the feasibility and acceptability of the trial [19, 21–23]. Furthermore, we used repeated measures of core outcome constructs in order to explore whether we can expect clinically relevant improvements in these measurements and included patient-reported outcome measures at baseline and week 12 in line with the core domain set for clinical trials of shoulder disorders recommended by the OMERACT group [53].

The study has limitations. First, because of the limited number of participants enrolled in this study it is difficult to determine whether the estimated rates addressing the feasibility outcomes (e.g. recruitment rate) are representative for the targeted population. Secondly, adherence to the self-managed exercises was lower than anticipated and because we did not include qualitative interviews, methods were not in place to explore the reasons in depth. A third limitation is that fidelity was measured in a limited way using only the physiotherapists’ journal notes. The intervention was delivered by experienced physiotherapists, which might have ensured high fidelity to the Ad-Shoulder protocol. Whether the fidelity will be equally high when implemented to other settings, for example in a multicentre trial, cannot be predicted from the present results. A fourth limitation is that we did not assess follow-up in a 12-month perspective as proposed for the main RCT. Therefore, the 3 months follow-up rate might be a bit optimistic of what we can expect at 12 months follow-up. Finally, we were not able to measure home exercise adherence objectively during the 12 weeks intervention period and the self-reported adherence might therefore be overestimated.

## Conclusions

The feasibility of recruitment and adherence to the self-managed exercise programme were both below the anticipated level. Valid objective physical activity data measures at baseline and week 12, follow-up methods, the physiotherapists’ fidelity to the intervention and patients reporting adverse events were all acceptable according to our progression criteria. Additional recruitment sites will be added to optimise recruitment and further intervention development including qualitative research is necessary to understand whether adherence to the self-managed exercise could be enhanced.

### Supplementary Information

**Additional file 1.** CONSORT 2010 checklist of information to include when reporting a pilot or feasibility trial.

**Additional file 2.** Content reporting of the self-managed exercise programme according to the Consensus on Exercise Reporting Template (CERT).

## Figures and Tables

**Table 4:**
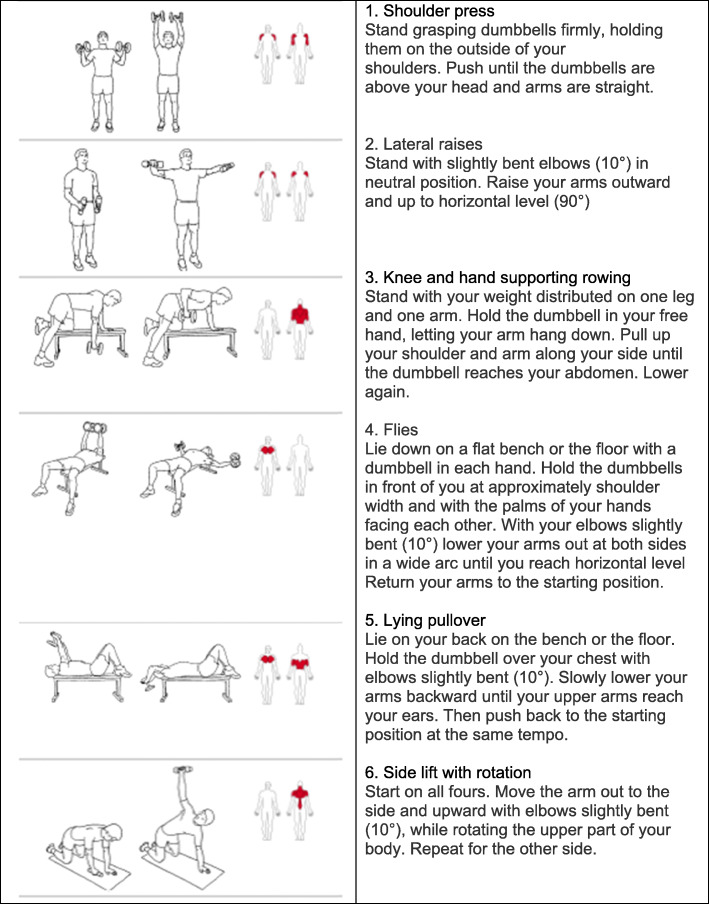
Self-managed exercise programme

## Data Availability

De-identified individual-patient data are available by contacting the corresponding author.

## Appendix 1

Description and operationalisation of the five core self-management skills

The core self-management skills are as follows:

1. Problem-solving

During the first session with the physiotherapist, the focus will be on actively involving the participant in how to keep a good performance level of exercises or how to improve their ability to exercise. The barriers for long-term adherence to exercises will be defined (problem definition), concrete solutions to overcome these barriers will be suggested based on shared decision-making (generation of possible solutions), and a brief set of 1–3 exercises (because of higher odds of adherence [54]) for the coming week will be agreed upon (solution implementation).

2. Decision-making

During the second session the decision-making process will be the main focus. The experiences from the first week(s) will be used to go into a more thorough discussion around how to implement a behaviour in line with adherence to shoulder exercises and how to become more confident in managing their shoulder pain by themselves. For example, the physiotherapist might consider that the participant needs more knowledge in order to meet the goal of long-term adherence to shoulder exercises. This can be related to topics such as how to deal with pain during or after the exercises, what is the optimal dosage of exercises/physical activity, and/or how to deal with fears and worries related to exercises and physical activities. The topic can also be how to organise the daily life in order to prioritise the shoulder exercises.

If pain is experienced while exercising, the participant will be told that as a rule of thumb it should be acceptable upon cessation or return to an acceptable pain level within 24 h. The participant will be encouraged to judge what is acceptable. If the participant experiences unacceptable pain during or after the exercise sessions, they will be advised to cut back on the exercise dosage and try to find a comfortable exercise level, stick to this for 1 or 2 weeks and add to it by 10 to 20% every 7 to 14 days. We will also explore the patients’ perception about exercising discussing aspects, such as “What do you think will happen when you perform this exercise? Do you think this exercise is dangerous for you?” [55].

This part also ensures that the intervention will be personalised. Based on the assessment and the comprehensive baseline questionnaire the management will emphasise key issues, such as graded exposure to feared movements for patients with kinesiophobia and/or low pain self-efficacy, discussing the importance of physical activity for patients not meeting current physical activity recommendations (< 150 min moderate activity per week), managing poor sleep, dealing with depression, anxiety and low mood and how to manage pain “flare-ups”.

3. Resource utilisation

This skill is related to teaching the participants in how to use available resources that might help them stay adhered to shoulder exercises and to reconceptualise unhelpful thoughts, pain beliefs and behaviours. For this specific study, we will encourage the participant to identify beneficial resources in their local environment, such as a local gym where they can exercise. The patients were also encouraged to do web lessons to learn more about the biopsychosocial nature of pain at retrainpain.org and watch a short video about pain (https://www.youtube.com/watch?v=E9tVWoRhPKU, available in Norwegian and English). The resources were utilised after the first session and the patients’ interpretation of these resources were discussed with the physiotherapist one-to-one in the following session.

4. Forming of a patient/health care provider partnership

The forming of a participant/physiotherapist partnership will include goal setting based on patients’ personal preferences by using the Patient Specific Function Scale. The patients will also be able to contact their physiotherapist when they are uncertain about how to manage their shoulder pain by themselves. To strengthen the therapeutic alliance, we will also allow patients to tell their story, provide emotional support and chat with the patient in a friendly manner and to motivate and show encouragement [56–59].

5. Taking action

Taking action reflects skills that are involved in learning how to change a behaviour. For all participants in the current study, an action plan for the next 1–3 weeks will be worked out, together with the patient at the first consultation. The action plan will contain information about the time points for exercises and/or other physical activities, the amount (number and length of sessions) and modifications of the exercises based on individual pain acceptability (e.g. lateral raise up to 45°). In relation to self-management theory, the action plan needs to reflect something that the participant is fairly confident to accomplish. Level of confidence will be measured by asking the patient how confident they are that they will do what is described in the action plan. The participant will score their level of confidence on a numerical rating scale from 0 (totally unconfident) to 10 (totally confident). If the answer is 7 or higher, based on self-efficacy theory, there is a good chance that the action plan will be accomplished. If the answer is less than 7, the physiotherapist will encourage further problem-solving in order to make the plan more realistic and to avoid failure. This usually involved decreasing the number of exercises due to time constrains.

During the last individual sessions, a long-term action plan will be developed together with the patient. In this plan, physical activities that may replace or supplement the exercise programme is discussed. This plan will be strongly tailored to the individual and contain valued activities. The patient will be strongly encouraged to continue an active-lifestyle in relation to physical activity involving a varied use of the upper-extremities.

## Appendix 2

Self-managed exercise programme

The exercise programme is performed at home 3 times per week. You can choose to do 1–3 exercises according to your action plan. Rest 2 min between sets. All exercises should be performed in a slow manner. Two to 3 s up, 2–3 s down. If unacceptable pain is felt when exercising: Modify range of motion and/or resistance until the symptom response is acceptable.

If you feel uncertain about how to manage your shoulder pain or if you are experiencing unacceptable pain, please contact your physiotherapist for collaborative problem-solving.

Videos of the exercises: https://exorlive.com/video/?ex=80,79,6,97,157,159&culture=nb-NO

Illustrations used with permission from © ExorLive.com

## Appendix 3

Patient-reported outcome measures

Pain and disability was assessed using the Norwegian language version of the Shoulder Pain and Disability Index (SPADI). SPADI is a self-reported questionnaire for patients with shoulder pain. The questionnaire consists of 13 questions divided into two domains: pain (five items) and function (eight items) and scored on numerical scale from 0 (best) 10 (worst) with a score range from 0 to 100 points [35]. A higher score indicated worse shoulder pain and disability [35]. The MCID has been estimated to be 8–13.2 in the literature [52]. A Norwegian translated and adapted version of the SPADI has shown acceptable reliability, agreement and responsiveness [60, 61] and it is the proposed main outcome measure for the main randomised controlled trial (RCT).

The Hopkins Symptom Checklist-25 (HSCL-25) [46] will be used as a measure of emotional distress. The questionnaire aims to assess symptoms of anxiety, depression and somatisation. HSCL-25 is a shorter version of the Symptom Checklist 90 (SCL-90) and consists of 25 items that are rated from 1 (not at all) to 4 (very much). The score will be obtained by averaging the scores and ranges from 1 and 4. A maximum of five missing items will be accepted. A higher average score indicates a higher level of emotional distress. Expectations of recovery were assessed at baseline using a 4-point ordinal scale (complete recovery, much improve, slightly improve, no change/worse) [47].

Pain intensity during the last week was measured using the NPRS (ranging from 0 = no pain, 10 = the most intense pain imaginable) [38].

Self-efficacy (confidence to manage the shoulder problem) was measured using one modified question from the Musculoskeletal Health Questionnaire: “How confident have you felt about managing your shoulder pain by yourself (responses were not at all confident, slightly, moderately, very and extremely)?” [39]. The score ranges from 1 to 5 (extremely confident, very confident, moderately confident, little confident and not at all confident).

Pain self-efficacy was measured at baseline and week 12 using a 2-item questionnaire (PSEQ-2) by asking the patients’ two questions: how confident they are doing some form of work and to live a normal lifestyle at present, despite the pain (ranging from 0 = not at all confident, 6 = completely confident) [40]. A person with a score of 5 or less might be considered in need of help with their confidence in functioning in the presence of their pain, while a score of 8 or higher reflects a desirable level of pain self-efficacy or confidence in functioning in the presence of pain [40].

Patient Specific Function Scale (PSFS) is a patient-specific outcome measure where the patient is asked to name three activities which the patient find challenging or are not able to do because of their shoulder pain [36, 37]. Patients rated their ability to complete the activities on an 11-point scale at a level experienced prior to injury or change in functional status. “0” represents “unable to perform” and “10” represents “able to perform at prior to pain level”. PSFS has been found to have good validity, reliability and responsiveness among many different patient groups, including patients with upper extremity musculoskeletal problems [62], neck pain [37] and low back pain [36].

Work ability was measured with Working Ability Index [41] on a 11-point scale (0 = cannot work at all, 10 = working ability is best right now) [41].

Generic health-related quality of life was assessed by the EQ-5D-5L [71]. It evaluates 5 dimensions: mobility, self-care, activities of daily living, pain and anxiety and/or depression. For each dimension the patient describes three possible levels of problems (none, mild-to-moderate and severe). This descriptive system therefore contains 3^5^ = 243 combinations or index values for health status. Total score ranges from − 0.59 to 1, where 1 corresponds to perfect health and 0 to death. Negative values are considered to be worse than death.

Pain-interference with sleep was assessed by the Bergen Insomnia Scale, which consists of six items [43]. Scoring 3 or above on at least one of the first four items and scoring 3 or above on at least one of the last two items indicate the presence of insomnia.

Kinesiophobia was assessed using one question [44]: “How much ‘fear’ do you have that these complaints would be increased by physical activity?” (scores range from 0 = no fear, to 10 = very much fear).

The patient rated perception of change at week 12 was assessed on a 6-point Global Perceived Effect scale (GPE) [48]. The responses were: very much improve, much improve, slightly improve, no change, worse and much worse.

Patient-reported physical activity was assessed by 3 items from the Nord-Trondelag Health Study (HUNT) [45] regarding frequency, intensity and duration.

## Abbreviations

BESS: British Elbow and Shoulder Society
MET: Metabolic equivalent
NPRS: Numeric Pain Rating Scale
RCT: Randomised controlled trial
PSEQ-2: Pain Self-Efficacy Questionnaire 2-item
SPADI: Shoulder Pain and Disability Index
