# Supplementary material for: Adherence to self-managed exercises for patients with persistent subacromial pain: the Ad-Shoulder feasibility study
Source: Pilot Feasibility Stud. 2021 Jan 25;7:31. doi: 10.1186/s40814-021-00767-6 (PMC7831168; doi:10.1186/s40814-021-00767-6)
Supplement: Supplementary file 2 — Additional file 2. Content reporting of the self-managed exercise programme according to the Consensus on Exercise Reporting Template (CERT). [file 40814_2021_767_MOESM2_ESM.docx]

Content reporting of the self-managed exercise program according to the

Consensus on Exercise Reporting Template (CERT)

| 1 Detailed description of the type of exercise equipment | The exercises will be performed with hand  held dumbbells adjusted to each individual’s capacity and tolerance |
| --- | --- |
| 2 Detailed description of the qualifications, expertise and/or  training | The intervention was delivered by 2 experienced physiotherapists with a master’s degree (MSc), at least 4 years clinical practice and a special interest in shoulder pain |
| 3 Describe whether exercises are performed individually or in a group | The exercises are performed individually (“one-on-one”) |
| 4 Describe whether exercises are supervised or unsupervised;  how they are delivered | During the 1-5 consultations, the exercises are conducted in an outpatient clinic with one-to-one supervision by a physiotherapist. The physiotherapist provides guidance, motivation and feedback and modifies exercises as appropriate by changing the resistance or range of motion based on the patients’ pain acceptability. The home-based exercises are unsupervised. The patients can contact the physiotherapist by SMS, e-mail or phone if they feel uncertain about managing their symptoms until 3 months follow-up. |
| 5 Detailed description of how adherence to exercise is measured and reported | Adherence to exercises will be measured with a continuous exercise logbook. Adherence to the self-managed exercises is reported in the results |
| 6 Detailed description of motivation strategies | For this study, the forming of a participant/physiotherapist partnership will include shared goal setting based on patients’ personal preferences by using the PSFS, and collaborative problem solving. We will make action plans based on shared decision making and make sure the patients feel confident that the action plan will be accomplished. To strengthen the patient-therapist relation we will allow patients to tell their story, provide emotional support, chat with the patient in a friendly manner and to motivate and show encouragement. |
| 7a Detailed description of the decision rule(s) for determining  exercise progression  7b Detailed description of how the exercise program was  progressed | We recommended that 10% increase in load be applied when the individual can perform the current workload for two repetitions over the desired number and judges the symptom response as acceptable. If the patient experiences unacceptable pain during, after or 24 hours post exercising, we recommend reducing the load by 10% or until the symptom response is acceptable. |
| 8 Detailed description of each exercise to enable replication | See Appendix 3 with illustrations, descriptions and videos of all exercises |
| 9 Detailed description of any home programme component | Patients were asked to perform the self-managed exercises at least 3 times each week in addition to the supervised physiotherapy sessions |
| 10 Describe whether there are any non-exercise components | The physiotherapists will also focus on resource utilization (eg. pain information) to enhance the participants’ decision-making skills and cognitive reassurance to increase knowledge, self-efficacy and change illness perception/beliefs. |
| 11 Describe the type and number of adverse events that occur during exercise | Reported in results |
| 12 Describe the setting in which the exercises are performed | During the 1-5 consultations the exercises will be performed at an outpatient clinic in a gym and the home-based program will be performed at home or in a gym based on individual preferences. |
| 13 Detailed description of the exercise intervention | See appendix 1 |
| 14a Describe whether the exercises are generic (one size fits all) or tailored | The exercises are individually adapted to each person. A maximum of three exercises will be selected based on the patients’ preferences each time a new action plan is made and agreed upon. |
| 14b Detailed description of how exercises are tailored to the individual | The exercises are tailored if the patients’ experiences unacceptable pain during the exercise. This usually involves conducting the exercise in a more limited range of motion or by decreasing the resistance until the symptom response is acceptable. E.g. Abduction 0-45° instead of 0-90°. |
| 15 Describe the decision rule for determining the starting level | The dose was 3 sets of 12-15 repetitions maximum (RM) the first two weeks, 3 sets of 10 RM the next two weeks, 8 RM the next eight weeks, with the starting weight matched to the participant’s 12-15 RM if their pain level is acceptable when performing the exercises. |
| 16a Describe how adherence or fidelity is assessed/measured | The physiotherapists attended a one-day training session (8 hours) covering delivery of both exercise programs and receive the same manual as in this manuscript describing the intervention, to reinforce similar treatment administration. If the therapists were unsure about what to do, they were encouraged to contact DHM.  The physiotherapists documented every individual consultation in a logbook for the researchers to be able to monitor to what extent the intervention was delivered as planned. |
| 16b Describe the extent to which the intervention was delivered as planned | Reported in results |
